# Editorial: The Ecology of Plant Chemistry and How it Drives Multi-Species Interactions

**DOI:** 10.3389/fpls.2019.00967

**Published:** 2019-07-30

**Authors:** Massuo J. Kato, Tara J. Massad, Mariana A. Stanton, Daniel G. Vassão, Lydia F. Yamaguchi

**Affiliations:** ^1^Department of Fundamental Chemistry, Institute of Chemistry, University of São Paulo, São Paulo, Brazil; ^2^University of Memphis, Memphis, TN, United States; ^3^Department of Biochemistry, Max Planck Institute for Chemical Ecology, Jena, Germany

**Keywords:** chemical ecology, plant secondary metabolites, plant volatiles, pollination, structure activity analysis, multi-trophic interactions, biodiversity, specificity

In this Research Topic issue, we are pleased to present a diverse collection of papers illustrating the importance of chemical ecology for our understanding of ecosystem functioning in both natural and agricultural settings. The field of chemical ecology is quickly advancing as new tools in chemical analyses and molecular biology allow us to better appreciate the degree to which plant chemistry influences interspecific interactions. The papers presented here are an excellent representation of the application of these techniques and demonstrate how plant chemistry shapes interspecific interactions across different scales.

Several papers in this issue represent a field experiencing exciting developments—research on the biochemical mechanisms of the action of defense compounds and their fates within herbivores. Chemical structure is critical to the function of secondary metabolites, even within broadly defined groups of compounds. Monarch butterflies, for example, are shown in fascinating work by Petschenka et al. to have Na^+^/K^+^-ATPases with broader and overall higher resistance to a suite of cardenolides compared to the enzymes of less specialized herbivores, but the biological effects of cardenolides from *Asclepias* host-plants do not simply reflect their inhibitory activities on monarch enzymes *in vitro*. The effects of different side-chain structures within another class of chemical defenses, glucosinolates, were studied by Jeschke et al. The authors used isotopes to demonstrate that specialist herbivores avoid proper activation of these defenses, while these same compounds were fully activated in generalist herbivores and differentially inhibited their development. Vassão et al. further show that plant glycosidases, including some responsible for the activation of glucosinolates and of a diversity of other plant defenses from a wide variety of species, remain undigested by generalist insects, indicating strong selective pressures on plants to promote the continuous activation of their defense compounds.

A mechanistic understanding of plant defense and of the specificity with which plants respond to different herbivores was addressed by Schuman et al. The authors used transgenic lines of *Nicotiana attenuata* with reduced production of jasmonoyl isoleucine, a jasmonic acid (JA) derivative that regulates many plant responses to herbivory, to test the specificity of plant resistance mechanisms. Elicitation by JA and its derivatives allows for plasticity in direct and indirect responses of plants to damage, as indicated by metabolomic analyses of volatiles and water-soluble compounds from experimentally treated plants. Moreover, a meta-analysis in this issue (Mundim and Pringle) examines above- and belowground changes in primary and secondary metabolites in response to water stress and herbivory, addressing long-debated questions about growth-defense trade-offs with the fine-grained detail offered by metabolomics. They further suggest more realistic studies of the effects of multiple, co-occurring stressors on plants are needed.

Plant volatiles are deservedly highlighted in this issue, reflecting their considerable interest to chemical ecology as we continue to learn about the roles they play as the first line of communication between plants and insects. Borges discusses research on volatiles and galling insects, stressing that the topic is understudied and requires further attention. New discoveries in pollination biology are brought to light by Wong et al. and Wong et al. whose careful study of orchid mimicry of female thynnine wasp sex pheromones demonstrates how developmental and tissue-specific transcriptional changes in orchid flowers allow orchids to successfully lure male pollinators to their flowers at just the right time. Suggestions for studies on the evolution and biochemical production of orchid scents used in deceptive pollination that can be applied to other non-model organisms are also presented by Wong et al. In addition, a study describing guarana flower pollination by Krug et al. shows that nocturnal bees are attracted by volatile blends that differ in composition from those released during the day.

At a larger scale, our understanding of tropical diversity is increasingly improved by chemical ecology, and some excellent papers in this issue demonstrate the importance of plant chemical diversity for tropical forest diversity. Vleminckx et al. show that secondary metabolites are less similar than expected by chance in co-occurring congeners, suggesting herbivory selects for chemical diversity. Endara et al. further show that a phylogeny of specialist sawflies matches plant chemotypes more closely than host plant phylogeny. The response of the third trophic level to chemical diversity was analyzed by Slinn et al. These authors used an invaluable 19-year-long database to determine that phytochemical diversity affects larval immunity against parasitoids in specialist but not generalist herbivores. This result differed between two tropical forests, however, and the authors open the door to further questions about the scale of these patterns and how the degree of herbivore specialization affects them.

Several of the articles in this edition highlight the value of considering chemically mediated interactions in applied arenas and indicate directions for future research that examines chemical ecology and tri-trophic interactions in complex agroecosystems. Addressing applications of chemical ecology to agriculture from the bottom-up, Blubaugh et al. report that rhizobium-inducing soil bacteria affect aphid growth and parasitism, underscoring the complexity of chemically-mediated trophic interactions. Furlong et al. and Silva et al. both discuss the role plant volatiles could play in improving agriculture. Silva et al. note that volatiles should receive more attention in studies targeting pollination and biocontrol, while Furlong et al. suggest that the failure to successfully translate laboratory tests of volatiles that attract parasitoids to field-level applications for improved biocontrol is due to the complexity of these interactions and the ability of parasitoids to discern between reliable and unreliable signals. Lastly, herbivore host-use is analyzed by Gallinger and Gross who show that psyllid agricultural pests that host-switch during their life cycle from fruit crops to pines are able to feed on but not complete their development on pines due to their phytochemistry.

We are excited to share this representative selection of fascinating work in the growing body of research linking plant chemistry to the ecology and evolution of multi-trophic interactions.

We hope you will enjoy these articles and that they will inspire further advances in the study of chemical ecology.

## Author Contributions

MK coordinated the editing for the Research Topic, contributed to the proposal of the Research Topic subject, and edited manuscripts. TM, DV, MS, and LY contributed to the proposal of the Research Topic subject and edited manuscripts.

### Conflict of Interest Statement

The authors declare that the research was conducted in the absence of any commercial or financial relationships that could be construed as a potential conflict of interest.

